# In-Silico Characterization of Glycosyl Hydrolase Family 1 β-Glucosidase from *Trichoderma asperellum* UPM1

**DOI:** 10.3390/ijms21114035

**Published:** 2020-06-04

**Authors:** Mohamad Farhan Mohamad Sobri, Suraini Abd-Aziz, Farah Diba Abu Bakar, Norhayati Ramli

**Affiliations:** 1 Department of Bioprocess Technology, Faculty of Biotechnology and Biomolecular Sciences, Universiti Putra Malaysia, Serdang 43400 UPM, Selangor, Malaysia; mfarhan@unimap.edu.my (M.F.M.S.); suraini@upm.edu.my (S.A.-A.); 2 School of Bioprocess Engineering, Universiti Malaysia Perlis, Kompleks Pusat Pengajian Jejawi 3, Arau 02600, Perlis, Malaysia; 3 School of Biosciences and Biotechnology, Faculty of Science and Technology, Universiti Kebangsaan Malaysia, Bangi 43600 UKM, Selangor, Malaysia; fabyff@ukm.edu.my

**Keywords:** *Trichoderma asperellum*, β-glucosidase, glycosyl hydrolase family 1, in-silico analyses

## Abstract

β-glucosidases (Bgl) are widely utilized for releasing non-reducing terminal glucosyl residues. Nevertheless, feedback inhibition by glucose end product has limited its application. A noticeable exception has been found for β-glucosidases of the glycoside hydrolase (GH) family 1, which exhibit tolerance and even stimulation by glucose. In this study, using local isolate *Trichoderma asperellum* UPM1, the gene encoding β-glucosidase from GH family 1, hereafter designated as *TaBgl2*, was isolated and characterized via in-silico analyses. A comparison of enzyme activity was subsequently made by heterologous expression in *Escherichia coli* BL21(DE3). The presence of N-terminal signature, cis-peptide bonds, conserved active site motifs, non-proline cis peptide bonds, substrate binding, and a lone conserved stabilizing tryptophan (W) residue confirms the identity of *Trichoderma* sp. GH family 1 β-glucosidase isolated. Glucose tolerance was suggested by the presence of 14 of 22 known consensus residues, along with corresponding residues L167 and P172, crucial in the retention of the active site’s narrow cavity. Retention of 40% of relative hydrolytic activity on ρ-nitrophenyl-β-D-glucopyranoside (ρNPG) in a concentration of 0.2 M glucose was comparable to that of GH family 1 β-glucosidase (Cel1A) from *Trichoderma reesei*. This research thus underlines the potential in the prediction of enzymatic function, and of industrial importance, glucose tolerance of family 1 β-glucosidases following relevant in-silico analyses.

## 1. Introduction

*Trichoderma* sp. is a genus of fungi that has been the subject of several studies, ranging from phylogeny, distribution, defense mechanism, host interaction, production and secretion of enzymes, sexual development, and also responses to changes in the environment [1]. In particular, there has been extensive interest in its capacity for cellulase production, resulting in its application in biotechnology and subsequent industrial fields, such as bioethanol production from lignocellulosic materials, textile fields, and feed production [2,3]. Efforts on this front have involved *Trichoderma reesei*, making it a fungal model organism, of focus for both industrial and academic research teams [4]. 

Cellulases function by hydrolysis of β-1,4 linkages present within cellulose. In nature, complete degradation is obtained by the synergistic action of three separate types, namely (1) endoglucanases, (2) cellobiohydrolases, and (3) β-glucosidases [5,6]. From the surface of solid substrates, primary hydrolysis by endoglucanase and cellobiohydrolase lead to the release of soluble cellodextrins. Secondary hydrolysis then proceeds for the breakdown of cellodextrins such as cellobiose into glucose by β-glucosidases [6]. In the process of cellulose saccharification, occurrences of substrate and production inhibition are not uncommon. In substrate inhibition, this occurs on a two-domain structure found on cellobiohydrolase, of which for a given fixed enzyme load, rise in substrate concentration results in increase of time/distance necessary for cellobiohydrolases to act on the chain ends resulting from endoglucanase activity [7]. The presence of liberated cellobiose and monosaccharides may instead lead to product inhibition where cellobiose affects the active site of cellobiohydrolases by steric hindrance [8] and β-glucosidases are affected by the presence of glucose [9]. Hence, to achieve increased performance of hydrolysis, efforts have included balancing the enzyme cocktail and tailoring specific enzymes [10]. 

Among the three types involved, β-glucosidase has been focused on owing to several characteristics. In production terms, β-glucosidase is noticeable at much lower concentrations than endoglucanases and cellobiohydrolases, even from commercial cellulase producer such as *T. reesei* [11]. Unfavorable end product inhibition by glucose further exacerbates the problem, making it the rate-limiting enzyme, with impaired yields being a major obstacle for commercialization of cellulose hydrolysis [9]. 

Classification based on sequence identity and hydrophobic cluster analysis, as available in carbohydrate-active enzymes (CAZy) database has placed β-glucosidases in glycoside hydrolase (GH) families 1 and 3 [12]. Among biotechnology industries, the utilization of β-glucosidases with glucose tolerance and stimulation can improve the efficiency of substrate degradation and result in a reduction of production costs. Thus, the utilization of glucose-tolerant β-glucosidases has increased in interest in recent years. While the majority of β-glucosidases are sensitive to glucose, tolerance coupled with a stimulatory effect of the carbohydrate have been observed exclusively among GH family 1 (GH1) β-glucosidases [13]. Comparison of relative tolerance to that of GH family 3 (GH3) β-glucosidases has been extensive, ranging from tenfold to 1000-times fold higher. Thus, it has been suggested that GH1 β-glucosidases are more suitable for plant cell-wall saccharification in biotechnological applications [14].

Collection, retrieval and analysis of biological data using computational techniques, in a science known as bioinformatics, has grown significantly with increase in computational speed and memory storage capabilities. Major impacts of the field include automation of genome sequencing, integrated genomics and proteomics databases, genome comparisons to identify the genome function, as well as the automated derivation of metabolic pathways, the development of statistical techniques, and three-dimensional (3D) modeling of biochemical structures. While, in general, sequence reveals structure, which reveals function, the reality can differ where a similarity in protein structures may result in different functions, and adversely, proteins with different structures may exhibit similar functions. Understanding and proving such relationships would be essential for the spectrum of applications bioinformatics can apply to [15].

While *T. reesei* has been the model organism for utilization, comparative secretome analysis to *Trichoderma asperellum* following solid state fermentation on the same biomass substrate have shown *T. asperellum* to have higher enzymatic activities and increased abundance of main and side chain hemicellulases and β-glucosidases [16]. Hence, from local *T. asperellum* UPM1 strain, this work has sought to isolate the gene encoding GH1 β-glucosidase (*TaBgl2*) and following expression in *Escherichia coli* as a recombinant enzyme, relate the enzyme’s specificity and sensitivity to glucose to physicochemical characteristics, ancestral relationship, and structure determination at several levels, predicted prior via in-silico analyses. 

## 2. Results and Discussion

### 2.1. Nucleotide and Deduced Amino Acid Sequence of TaBgl2

By means of nucleotide sequence analyzed using Basic Local Alignment Search Tool (BLAST) (Supplementary Table S1), isolation and sequencing of cDNA obtained was found to code for β-glucosidase of GH1, hereafter designated as *TaBgl2*. Translation into the corresponding amino acids led to identification of an open reading frame (ORF) of 1398 bp, G-C content of 55.65%, with subsequent protein BLAST elucidating a β-glucosidase, 465 amino acids in length, weighing approximately 52 kDa. From subsequent protein sequence, BLAST analysis (Supplementary Table S2) was carried out to deduce the identity of the isolated protein. Highest homology was found to β-glucosidase isolated from *T. asperellum strain* CBS 433.97 (accession number: XP 024766195.1) with identities of 99%. Upon comparison to selected *Trichoderma* spp. β-glucosidases, TaBgl2 was shown to have comparable amino acid length (between 455 and 466 amino acids). These findings coupled with the high degree of identities, i.e., exceeding 90%, further affirms the identity of the protein.

### 2.2. Sequence Alignment and Conserved Motif Identification

**Figure 1:**
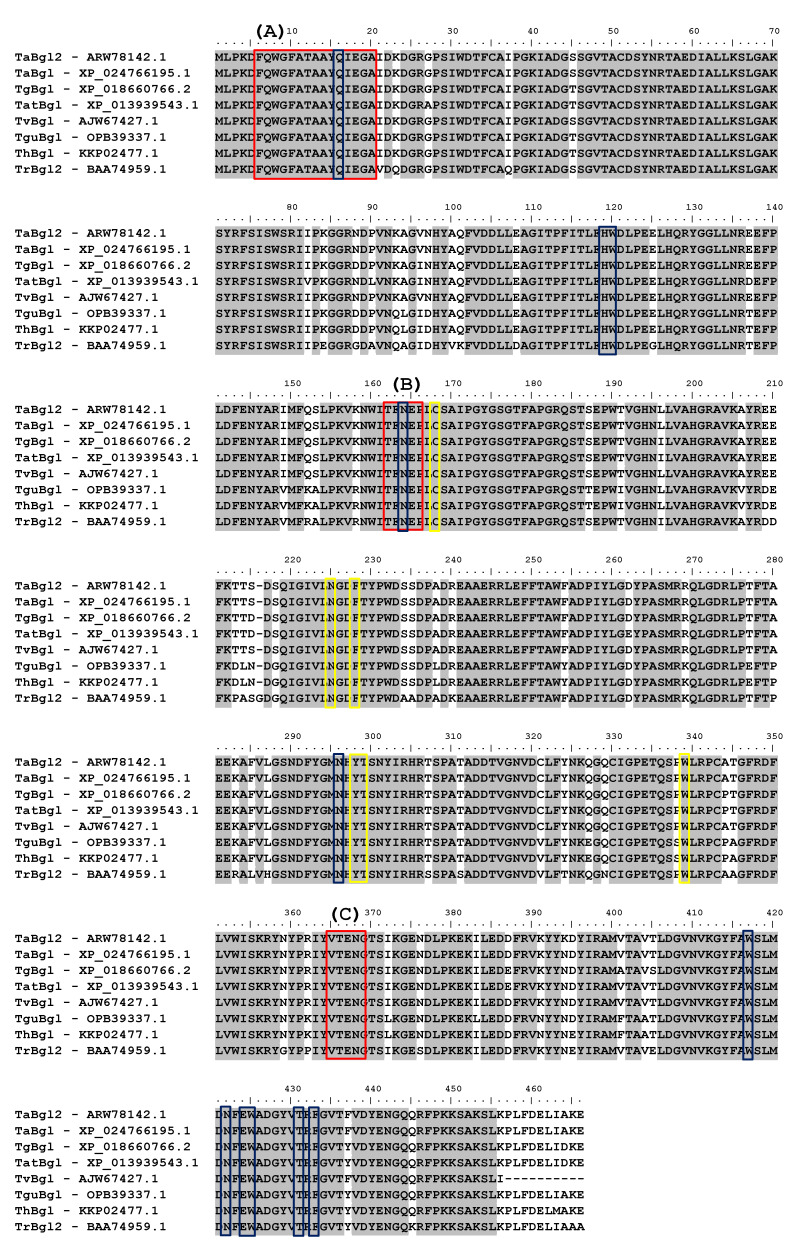
Sequence alignment of TaBgl2 with selected *Trichoderma* spp. β-glucosidase protein sequences (denoted by their enzyme abbreviations and NCBI/GenBank accession numbers) (TaBgl2, *T. asperellum* UPM1 Bgl 2 – ARW78142.1; TaBgl, *T. asperellum* CBS 433.97 glycoside hydrolase family 1 – XP_024766195.1; TgBgl, *T. gamsii* β-glucosidase – XP_018660766.2; TatBgl, *T. atroviride* IMI 206040 glycoside hydrolase family 1 – XP_013939543.1; TvBgl, *T. virens* β-1,4-glucosidase – AJW67427.1; TguBgl, *T. guizhouense* GH1 β-glucosidase Bgl2 – OPB339337.1; ThBgl, *T. harzianum* β-glucosidase – KKP02477.1; TrBgl2, *T. reesei* β-glucosidase – BAA74959.1). Highlighted motifs, in red boxes, include: (**A**) glycosyl hydrolase family 1 N-terminal signature, with (**B**) & (**C**) as catalytic site motifs. Boxed in blue and yellow are suggested glycone and aglycone binding residues, respectively.

Multiple sequence alignments (MSAs) have become a fundamental approach in molecular biology and bioinformatics research domains, including phylogenetic tree reconstruction, three-dimensional (3D) structure prediction, conserved regions identification, and elucidation of molecular function [17]. Hence, for purpose of conserved motif identifications, TaBgl2 was aligned to several β-glucosidases of known *Trichoderma* spp. origins (Figure 1). GH1 protein identity was suggested by the presence of two distinct motifs, namely N-terminal signature sequence with 15 amino acids in length (F-x-[FYWM]-[GSTA]-x-[GSTA]-x-[GSTA](2)-[FYNH]-[NQ]-x-E-x-[GSTA], present as FQWGFATAAYQIEGA in TaBgl2) [18], and two cis-peptide bonds between Ala-180 and Pro-181 and between Trp-416 and Ser-417 [19].

β-glucosidase identity was confirmed by the presence of two conserved motifs [20], TFNEP and VTENG, each containing the catalytic acid/base and nucleophile glutamate residues, E165 and E366, respectively. Identification of glutamate residues on GH1 β-glucosidase active site in TaBgl2 suggests as to the mechanism of activity, proceeding by the β-retaining mechanism [21] commonly found in other GH1 β-glucosidases. In addition, 22 residues constructing the entrance to the active site region were found along with 11 glycone binding residues, six aglycone binding residues [22], and a lone conserved tryptophan W338, stabilizing the aglycone moiety at the +1 subsite [14]. Matching of signature sequences based on models classifying proteins into families or for prediction of characteristic domains and functionally relevant sites allows for prediction of enzyme function [23].

As mentioned, a case can be made as to the possible glucose tolerance of GH1 β-glucosidases. Work by Mariano et al. [24] has highlighted 22 essential residues to confer this property of which 14 was identified with two being active site residues (E165, E366), eight glycone binding residues (H119, W120, N164, N295, W416, E423, W424, F432), and four aglycone binding residues (C168, N224, Y297, T298).

### 2.3. Secondary and Tertiary Structure Predictions

**Figure 2:**
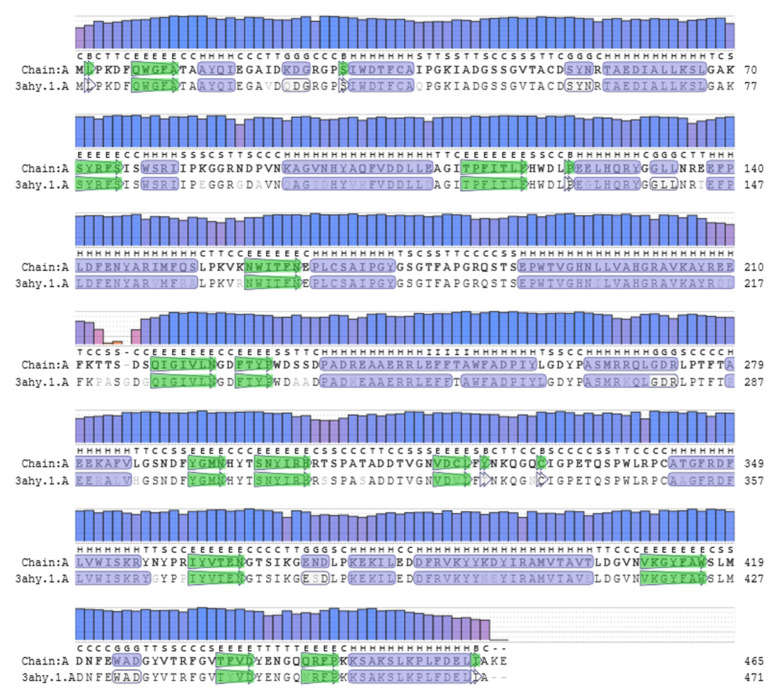
Alignment of TaBgl2 (represented as Chain A) to template *Trichoderma reesei* Bgl2 associated with Tris (3ahy.1). QMEAN values, displayed by bar height, function to quantify modelling errors and simultaneously estimate the expected model accuracy. α-Helix and β-sheets are colored as blue and green segments, respectively.

**Figure 3:**
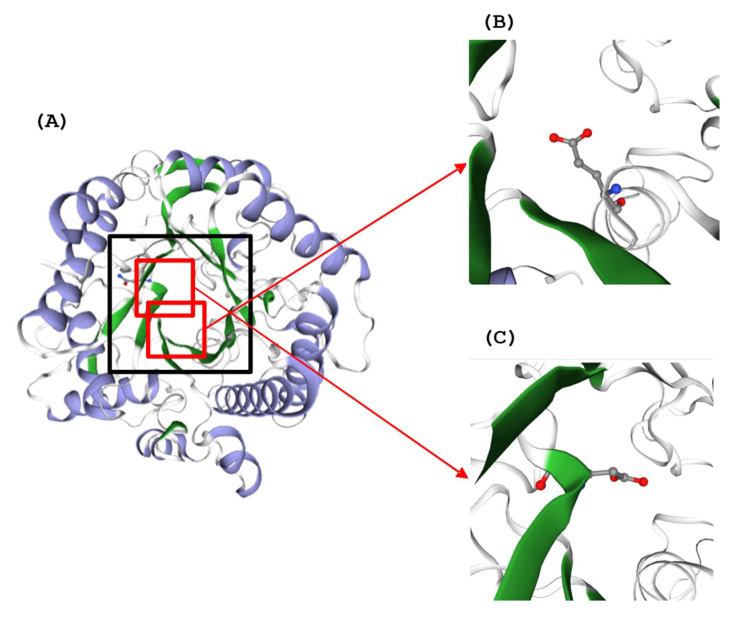
(**A**) Three-dimensional (3D) structure of TaBgl2 on the side of the active site entrance (boxed). Glutamate residues (E165 and E366) within catalytic motifs (TFNEP and VTENG) are as seen in (**B**,**C**), respectively. α-Helix and β-sheets are colored blue and green, respectively.

Secondary structure of TaBgl2 as well as selected β-glucosidase sequences of *Trichoderma* spp. were estimated using GOR IV tools (Table 1). The GOR method is a popular secondary structure prediction scheme, long established as the first implemented as a computer program. It functions by providing estimates of probabilities for three secondary structures at a given residue position, and has the advantage over nearest-neighbor and neural network-based methods by the identification of what is considered and neglected parameters for prediction [25]. To this, the GOR IV results suggest the dominance of random coils (54.41%–57.94%) among *Trichoderma* spp. β-glucosidases. *T. reesei* Bgl2 in complex with Tris (PDB accession number: 3AHY_A) [26] was used as a template, given a shared sequence identity of 90.06% and coverage of 1.0. Alignment of both sequences with the resultant protein structure was made (Figure 2 and Figure 3). TaBgl2 structure was observed to have the classical (α/β)8 triosephosphate isomerase (TIM) barrel fold. From this, an outer opening was created which the slot forms an active site cleft, with the catalytic motifs present opposite each other at the bottom of the active site, where a parallel β-barrel is formed from the β-sheets with α-helices located outside of the barrel [22,27,28].

Referring to Jeng et al. [26] as the source of the template structure TrBgl2, comparisons were also made to β-glucosidases from bacterium *Clostridium cellulovorans* and termite *Neotermes koshunensis*. To all proteins, active sites were found to form slot like clefts, between 15–20 Å in depth, positioned on connecting loops at the C-terminal end of β-sheets of the TIM barrel, surrounded by negatively charged residues. Specifically, for TrBgl2, the catalytic acid/base of TrBgl2 of Glu165 is located on the TFNEP motif at the end of the β-strand 4, while the catalytic nucleophile of Glu367 is located on the VTENG motif at the end of β-strand 7. Both residues are positioned as predicted for TaBgl2.

SWISS-MODEL analysis also suggested as to the presence of a non-proline cis-peptide bond, located between Trp416 and Ser417. As is common for residues involved in non-proline cis peptides bonds, the main-chain dihedral angles of both residues were found located on the β-region of a Φ/Ψ plot on a Ramachandran plot [29]. While regarded as rare, presence of the bond close to the active site supports several works on carbohydrate-binding or processing proteins such as *lac*Z *β*DG structure from *Escherichia coli* [30] and *β*-D-galactosidase from *Paracoccus* sp. 32d [31], suggesting presence of the bond to be a characteristic for glycosyl hydrolase proteins.

Concerning the argument proposing the tolerance of glucose to GH1 β-glucosidases, focus may be placed on the shape and the electrostatic properties of the entrance to the active site. Relative higher glucose tolerance of GH1 β-glucosidases can be correlated to the active-site accessibility, of which the cavities are deeper and narrower compared to the shallow ones present in GH3 β-glucosidases. With protein structures elucidated and knowledge of the conserved residues in TaBgl2, the importance of two additional key residues (W169 and L174 on *Thermoanaerobacter brockii* β-glucosidase) has further been argued to be essential for glucose tolerance [14]. These residues help in reducing enzymatic inhibition by retaining active site width, keeping a narrow cavity by means of imposing restrictions at the +2 subsite, thus limiting access of glucose to the +1 subsite.

Comparison of known glucose tolerant β-glucosidases from *Humicola insolens* (HiBG) and from a compost metagenomic library (GenBank accession No. HV538882) (Td2F2) where these residues are present, highlighted instead to their corresponding absence in TaBgl2, to be instead replaced as L167 and P172. Nevertheless, it must be highlighted that the 14 conserved residues and two key residues mentioned are identical to that of a previously elucidated glucose tolerant β-glucosidase from *T. reesei*, known as Cel1A [32]. Hence, a comparison of enzyme activity between novel TaBgl2, following heterologous expression, to the aforementioned protein, is hereby argued to provide a basis in demonstrating the feasibility of applying in-silico bioinformatics analyses for prediction of enzyme activity, specifically in the presence of glucose inhibitors.

### 2.4. Physicochemical Characterization

Physicochemical properties (isoelectric point (pI), number of positive and negative amino acids (R+/−), extinction coefficient (EC), instability index (II), aliphatic index (AI), grand average of hydropathicity (GRAVY) and total number of atoms (TNA)) for β-glucosidases from different *Trichoderma* spp. were determined (Table 2). The isoelectric point of a protein indicates the net charge of a given protein (positive or negative) under physiological conditions, which in turn acts as a good indicator for the protein’s solubility at a given pH. A suggested pI value of 5.55 suggests for TaBgl2 to be considered an acidic protein [33]. By comparison, the value is also close to the pI range of the other *Trichoderma* spp. β-glucosidases compared (5.10–5.53) and to β-glucosidases from *T. reesei*, of which the range given was between 4.4 and 8.7 [20].

The instability index functions as a protein primary structure-dependent approach for in vivo protein stability predictions. Here, the instability index value of TaBgl2 which was given as 31.25 is again comparable to the range of the other *Trichoderma* spp. β-glucosidases compared (25.55–32.33), all of which suggests as to their stability, given values are less than 40 [34]. The aliphatic index in turn describes the relative volume of a protein occupied by its aliphatic side chains in which the higher the value, the more thermally stable the protein is predicted to be. The aliphatic index value of TaBgl2 was found to be 69.27, comparable to that of the other *Trichoderma* spp. β-glucosidases mentioned (68.65–72.19) and suggests the thermal stability of the enzyme [35].

With GRAVY value for TaBgl2 being negative at −0.450, similar consistent negative GRAVY values for *Trichoderma* spp. β-glucosidases meanwhile collectively suggests as to the high possibility for aqueous interactions [36]. The total number of atoms for the *Trichoderma* spp. β-glucosidases were between 7143 and 7341, with TaBgl2 containing 7319 in total, further suggests as to its comparable value. Screening through the amino acid compositions (%) of the selected *Trichoderma* spp. β-glucosidases (Table 3), indicates glycine to be consistently the dominant amino acid, ranging from 7.7% to 9.0%.

ProtScale tool analysis in turn led to prediction of minimum and maximum hydrophobic positions and scores for each β-glucosidase predicted (Table 4). Hydropathy plots constructed by ProtScale tool (Supplementary Figure S1) indicated to the absence of a 19-residue segment averaging greater than +1.6 for all β-glucosidases, which in turn suggests low probability for a membrane-bound segment of the protein, given that membrane spanning sequences are distinguishable to those that pass through a protein’s center, by the former’s higher hydropathy [37]. 

### 2.5. Protein Localization

**Figure 4:**
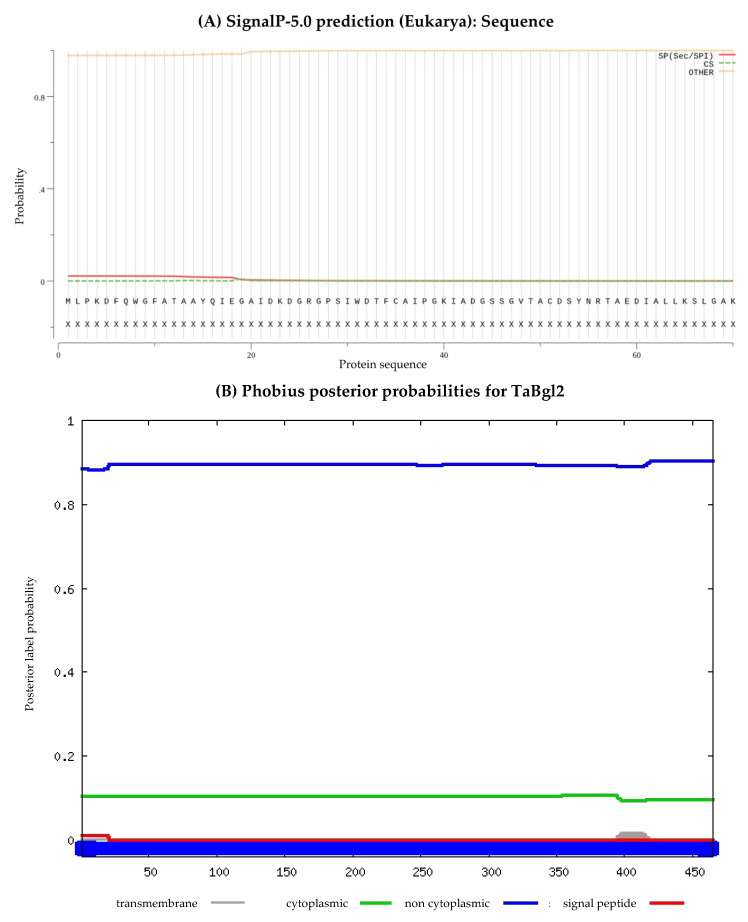

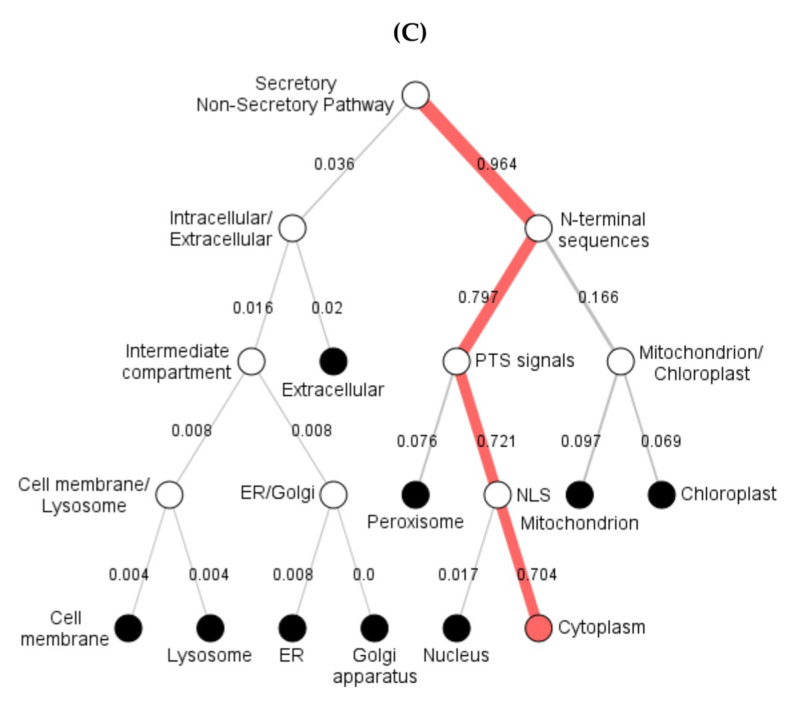
The subcellular position of predicted TaBgl2 protein presented by (**A**) SignalP 5.0, (**B**) Phobius and (**C**) DeepLoc-1.0 software.

In predicting the presence of signal peptides, SignalP 5.0 and Phobius was utilized (Figure 4A,B, respectively), with the likelihood of signal peptide at 0.0213 suggested by the former. In terms of protein localization, the relatively low probability value suggests the inability to detect for presence of any signal peptide to be non-cytoplasmic [38], a conclusion further supported by Phobius analysis. MitoProt analysis further indicated probability of export to mitochondria to be at 7.08%. Subcellular localization was predicted from PSORT II and DeepLoc 1.0 (Figure 4C). The resultant values of 0.565 and 0.6021 obtained respectively suggest as to the highest probability of the protein to be present predominantly in the cytoplasm, with the latter value suggestive of the protein being in soluble form (Supplementary Table S3).

Such positioning may offer insight as to the function of TaBgl2 in host *T. asperellum* UPM1, as implied from other similarly located β-glucosidase from *Trichoderma* sp. In a previous study, a GH1 β-glucosidase designated as Cel1A from *T. reesei* which also exhibited intracellular expression was argued to be involved in the induction of cellulase genes by lactose [39]. Guo et al. [32] further proposed that the protein was involved in the generation of sophorose by means of transglycosylation activities, again for the purpose of cellulase induction. To account for the intracellular expression, focus was placed into the N-terminal signal sequence. In a comparative study on the N-terminal amino acid sequences of extracellular *Humicola grisea* BGL4 and intracellular *T. reesei* BGLII, the positioning of aspartate residue was considered to be key. As an acidic residue, aspartate is not often found in the amino terminal of signal sequences, having instead one or two positively charged amino acids, such as arginine, lysine or histidine [40]. 

**Figure 5:**
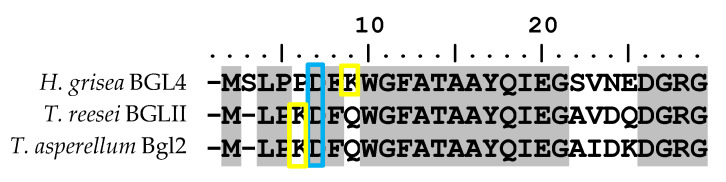
Alignment of N-terminal amino acid sequences of *Humicola grisea* Bgl4, *Trichoderma reesei* BglII and *Trichoderma asperellum* Bgl2 (TaBgl2). Boxed in blue and yellow are aspartate and lysine residues, respectively.

To the purpose of affirming this hypothesis, alignment of N-terminal amino acid sequences of *H. grisea* BGL4, *T. reesei* BGLII and TaBgl2 was done (Figure 5). In both *T. reesei* BGLII and *H. grisea* Bgl4, lysine (K) was found to be present near to the aspartate (D) residue. However, a noticeable difference can be seen in their relative positioning, of which in *H. grisea* BGL4, the lysine is two residues after aspartate, while in *T. reesei* BGLII, the lysine is located exactly prior to the aspartate, similar to that seen in TaBgl2. Thus, it has been suggested that not only is the presence of key amino acids critical, the difference in relative position of lysine to the aspartate residue could also account for the effectiveness of the signal sequence. 

### 2.6. Phylogenetic Analysis

**Figure 6:**
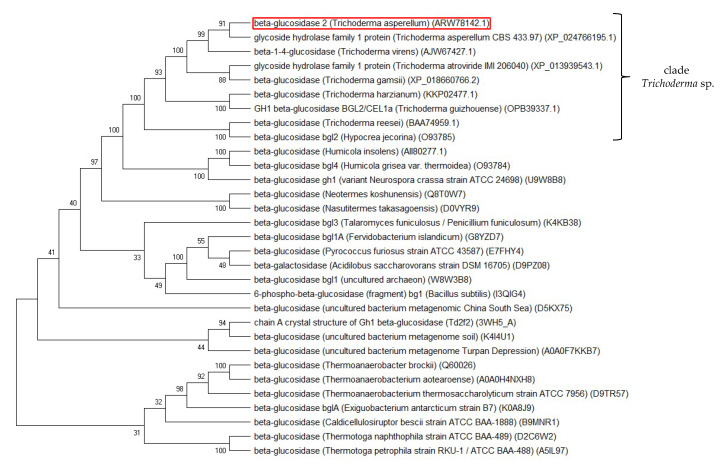
Neighbor-joining phylogram of selected homologous glucose tolerant β-glucosidases and TaBgl2 (ARW78142.1; boxed red) generated via MEGA X. The bootstrap consensus tree is inferred from 1000 replicates, with the confidence values shown next to the branches.

Phylogeny analysis via a neighbor-joining method to seven β-glucosidases of known *Trichoderma* spp. origins and several known glucose tolerant β-glucosidases was carried out (Figure 6). TaBgl2 was positioned to be clustered with putative β-glucosidases from *Trichoderma* spp., with closest relationship to GH1 protein from *T. asperellum* 433.97 (XP_024766195.1). Of further note is the further distance between TaBgl2 to known glucose tolerant β-glucosidases, which is suggestive of a lower glucose tolerance for the protein isolated.

### 2.7. Heterologous Expression of TaBgl2 in E. coli

**Figure 7:**
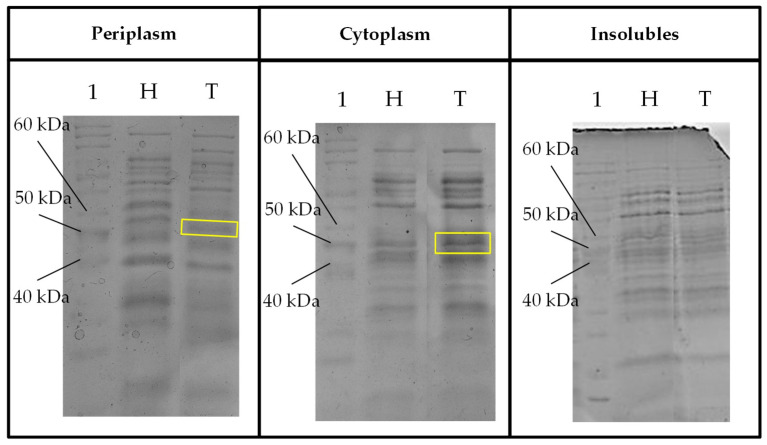
Sodium dodecyl sulfate-polyacrylamide gel electrophoresis (SDS-PAGE) profile of fraction samples (periplasm, cytoplasm and insoluble) for expressed protein visualization from crude protein extracts of host *Escherichia coli* and transformant TaBgl2 carrying plasmid pET-20b(+)-bgl2. Protein sizes were compared to bands separated from PageRuler™ Unstained Protein Ladder (Lane 1, in all gels) with a ~50 kDa band corresponding to the deduced recombinant TaBgl2 in the *E. coli* BL21(DE3) transformant (boxed yellow). H, Host; T, Transformant. Biological sample triplicates were prepared for all SDS-PAGE profiles.

As previously mentioned, comparable structural features and motifs of TaBgl2 to Cel1A elucidated [32] have been identified. Hence, following heterologous expression, it is hereby argued to provide a basis in demonstrating the feasibility of applying in-silico bioinformatics analyses for the prediction of enzyme activity, specifically in the presence of glucose inhibitors. As per Genscript source calculations, codon optimization on TaBgl2 gene resulted in increased codon adaptation index from 0.68 to 0.89 (Table 5). Both host and TaBgl2 transformants were then subjected to cultivation and from crude protein samples, heterologous protein production was suggested by the presence of protein bands of approximately 52 kDa in size from both periplasmic and soluble cytoplasmic fractions (Figure 7). In the absence of post-translational modification, the presence of bands corresponding to the 50 kb band of the protein ladder utilized are suggestive of the theoretical protein size of 52 kb calculated for TaBgl2 and falls within the molecular weight range (between 51.6 and 52.9 kDa) of β-glucosidases from other *Trichoderma* spp. (Supplementary Table S2).

**Figure 8:**
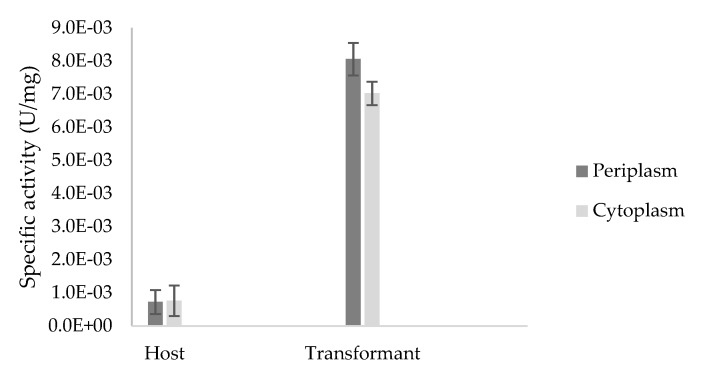
Specific activity (U/mg) of crude enzyme extracts from the periplasmic and cytoplasmic fractions of *Escherichia coli* BL21(DE3) host and transformant TaBgl2 carrying plasmid pET-20b(+)-bgl2 with ρ-nitrophenyl-β-d-glucopyranoside (ρNPG) used as a substrate. Biological sample triplicates were prepared for analysis and the specific activities in the periplasm and cytoplasm of host and transformant were shown to be significant (*p* < 0.05), as per Analysis of Variance (ANOVA) Single Factor analysis.

Subsequent β-glucosidase activity assay on ρNPG substrate was carried out on both fractions from host and transformant (Figure 8). Enzymatic β-glucosidase activity assay was significantly 11.1-fold higher in the transformants compared to the host, supporting the successful expression of heterologous TaBgl2 from transformants as opposed to that present in host strain. Between the transformants, periplasmic fraction demonstrated the highest specific enzyme activity (8.1 × 10^−3^ U/mg) compared to the cytoplasm (7.0 × 10^−3^ U/mg). This is expected given the presence of the N-terminal pelB signal peptide, directing protein expression to the periplasm. 

**Figure 9:**
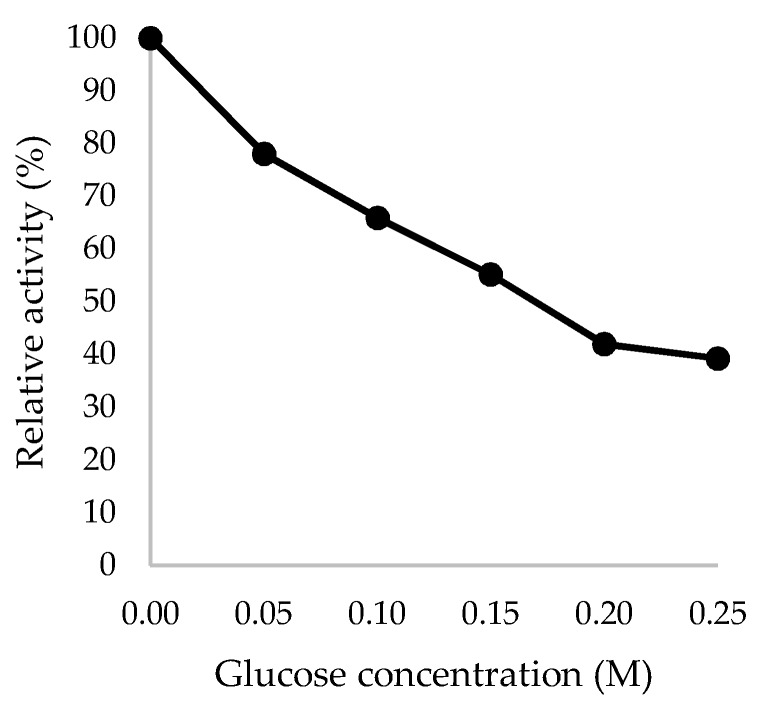
Glucose tolerance of recombinant TaBgl2 against glucose concentrations up to 0.25 M, expressed in relative activity (%). Biological sample triplicates were prepared for analysis.

Additionally, while transformants exhibit higher specific activity across both fractions, the basal activities of β-glucosidase observed in host samples are possibly attributable to the expression of *bglX*, a β-d-glucosidase located on the periplasm. The inherent gene coding for *bglX* is located adjacent to the *did* gene on the *E. coli* chromosome, with the 2.6 kb DNA sequence fragment revealing an ORF encoding a protein with 765 amino acids in length [41]. In the presence of glucose, relative enzyme activity of heterologous TaBgl2 was retained by as much as 40% in glucose concentration of up to 0.2 M (Figure 9). This was found to be comparable to that of Cel1A from *T. reesei*, which suggests the feasibility of utilizing in-silico analyses and subsequent heterologous expression for the prediction of glucose tolerance.

## 3. Materials and Methods

### 3.1. Strains and Plasmid Vector

*Trichoderma asperellum* UPM1 was a local isolate derived from rotten oil palm fruit bunch with species identification carried out via 18S rDNA methodology [42]. *Escherichia coli* BL21 (DE3) and plasmid pET-20b(+) were used for the expression of heterologous TaBgl2, targeted to the periplasm (Genscript, Piscataway, NJ, USA).

### 3.2. Isolation of TaBgl2 Nucleotide Sequence

Stock cultures of *T. asperellum* UPM1 were grown on potato dextrose agar (PDA) at 30 °C for 7 days prior to spore resuspension in 5% Tween 80. Mycelial hyphae were cultivated via submerged state fermentation in Mendel’s media (2 g/L potassium dihydrogen phosphate, 0.3 g/L magnesium sulphate heptahydrate, 0.3 g/L calcium chloride dihydrate, 1.4 g/L ammonium sulphate, 0.75 g/L peptone, 0.002% Tween 80) with trace element (0.005 g/L ferrous sulphate, 0.016 g/L manganese (II) sulphate, 0.014 g/L zinc sulphate heptahydrate, 0.002 g/L cobalt (II) chloride). Carboxymethylcellulose (CMC) at 1% was used as a carbon source and 1% β-lactose as an inducer at pH 5 with 10^6^ conidia per flask for inoculation [43]. Harvest of mycelia was done by vacuum filtration [44] using pre-sterilized nylon filters (GE Healthcare Life Sciences, Buckinghamshire, UK).

Total RNA was prepared from 0.1 g of wet mycelium by grinding using liquid nitrogen as per protocol provided with the GeneMATRIX Universal RNA Purification Kit (EURx, Gdansk, Poland). First strand cDNA was amplified using RevertAid First Strand cDNA Synthesis Kit (Thermo Scientific, Waltham, MA, USA) with 1 μg of total RNA as a template. Partial *TaBgl2* sequence amplification was carried out using degenerate primers (Table 6). PCR reactions proceeded with initial denaturation at 94 °C for 2 min, and 35 cycles of 94 °C for 30 s, 52.6 °C for 30 s, and 72 °C for 1 min, followed by final extension at 72 °C for 10 min [45]. PCR amplification consisted of 2.0 μL of 10× *Taq* buffer with KCl, 1.5 mM MgCl_2_, 0.8 mM deoxyribonucleoside triphosphate (dNTP) solution, 0.8 μM each of forward and reverse primers, and 2.5 × 10^−2^ U/μL of *Taq* DNA polymerase to a total volume of 20 μL. Polymerase, buffer, MgCl_2_ and dNTP were all sourced from Thermo Fisher Scientific, Waltham, MA, USA.

Sequencing procedures were carried out by Apical Scientific Sdn. Bhd., Malaysia. Following sequencing, specific primers were designed (Table 7) for use with rapid amplification of cDNA ends (RACE) PCR as suggested by SMARTer^®^ RACE 5′/3′ Kit User Manual (Clontech Laboratories Inc., Mountain View, CA, USA) until nucleotides coding for *TaBgl2* gene sequence was obtained. From elucidated nucleotide sequence, *TaBgl2* gene sequence was translated using ExPASy Translate Tool (https://web.expasy.org/translate/) using standard genetic code, for identification of ORF. The gene sequence was submitted to GenBank with the accession number KX772748.1 with the translated protein sequence accessible on NCBI through accession number ARW78142.1.

### 3.3. Sequence Alignment and Conserved Motif Identification

Homologous β-glucosidase protein sequences from several *Trichoderma* spp. and the degrees of identities were identified via BLASTp (protein) available from National Center for Biotechnology Information (NCBI). BioEdit Sequence Alignment Editor version 7.2.6 (North Carolina State University, Raleigh, NC, USA) [46] and ExPASY-PROSITE (SIB Swiss Institute of Bioinformatics, Geneva, Switzerland) [47] were then utilized for amino acid sequence alignment and the identification of conserved motifs, respectively.

### 3.4. Secondary and Tertiary Structure Predictions

The construction of secondary and tertiary protein structures from primary protein sequence template was conducted using SWISS-MODEL (https://swissmodel.expasy.org/). Structure analyses were further supported with GOR IV tool [25] for the determination of α-helix, β-sheet, turns, and loops.

### 3.5. Physicochemical Characterization

Physicochemical properties such as pI, number of positive and negative charged residues, extinction coefficient, instability index, aliphatic index, GRAVY, amino acid composition of β-glucosidases retrieved were computed using ProtParam tool (SIB Swiss Institute of Bioinformatics, Geneva, Switzerland) [36]. The determination of pI is by the pK value of protein during protein migration under denaturation conditions [48]. The concentration of purified protein in sample is determined by extinction coefficient value [49]. Protein stability is predicted from the instability index with values smaller than 40 suggesting for stable proteins and the proteins are regarded as unstable at values above 40 [50]. The volume occupied by aliphatic amino acids side chains (alanine, valine, leucine, and isoleucine) relative to total volume occupied is indicated by the aliphatic index and determines a globular protein’s thermostability [51]. GRAVY, which indicates the ratio of sum of hydropathy values of all amino acids to total number of residues in sequence, suggests as to the hydrophilicity or hydrophobicity of the protein [49]. Hydrophobicity scores, positions, and hydropathy plots on Kyte and Doolittle scale for all sequences were predicted by ProtScale tool (SIB Swiss Institute of Bioinformatics, Geneva, Switzerland) [51].

### 3.6. Protein Localization

Several software tools including SignalP 5.0 (Technical University of Denmark, Kgs Lyngby, Denmark), Phobius (Karolinska Institutet, Stockholm, Sweden), MitoProt (École Normale Supérieure, Paris, France), PSORT II (The University of Tokyo, Tokyo, Japan) and DeepLoc 1.0 (Technical University of Denmark, Kgs Lyngby, Denmark) were used for the prediction of signal peptide, cleavage site locations, subcellular localization, and the cell compartment, of which TaBgl2 is expected to act [52,53,54,55,56].

### 3.7. Phylogenetic Analysis

Molecular Evolutionary Genetics Analysis (MEGA) X software tool version 10.0.4 (Pennsylvania State University, PA, USA) was used to determine phylogeny of TaBgl2 to selected β-glucosidase sequences. following multiple sequence comparison by log-expectation (MUSCLE) alignment, the neighbor-joining method was used for inferring evolutionary history, with assessment of ‘confidence’ of the results done through 1000 bootstrap replicates [57].

### 3.8. Heterologous Expression of Recombinant TaBgl2

Codon optimization, subsequent synthesis, cloning into plasmid pET-20b(+) and transformation of *TaBgl2* gene into host *Escherichia coli* BL21(DE3) were carried out by GenScript (Hong Kong, China) Limited (Hong Kong, China). Restrictions sites *Bam*HI and *Hin*dIII on multiple cloning site of plasmid pET-20b(+) were selected for insert cloning with the resultant plasmid annotated as pET-20b(+)-*bgl2*. Transformants were confirmed by selective growth on LB agar plates supplemented with 100 μg/mL ampicillin (Amresco, Solon, OH, USA), with subsequent sequencing procedures using T7 terminator primer on extracted plasmid template that were carried out by Apical Scientific Sdn. Bhd. (Seri Kembangan, Selangor, Malaysia).

*E. coli* strains harboring expression vector for TaBgl2 were grown in 50 mL of Terrific Broth liquid medium (24 g/L yeast extract, 12 g/L tryptone, 4 mL/L glycerol, 100 mL/L potassium phosphate solution containing 0.17 M KH_2_PO_4_ and 0.72 M K_2_HPO_4_) supplemented with 100 μg/mL ampicillin (Amresco, Solon, OH, USA) in 250-mL flasks on a rotary shaker (150 rpm) at 37 °C. Cultivation then continued for 14 h with temperature reduced to 25 °C once absorbance at 660 nm reached 0.7. Resultant cultures were later separated, with the fractions designated as ‘extracellular’, ‘periplasm’, and ‘cytoplasm’, respectively.

### 3.9. Preparation of Extracellular, Periplasmic and Cytoplasmic Protein Fractions

Extracellular fraction was separated by centrifugation at 4 °C, 1880× *g* for 12 min [58]. The resultant sediment was then re-suspended in 20 mL of 30 mM Tris-HCl, pH 8 with 20% sucrose (Sigma-Aldrich, St. Louis, MI, USA) prior to addition of 1 mM ethylenediaminetetraacetic acid (EDTA), agitated lightly for 10 min at room temperature. Centrifugation was again applied at 4 °C, 10,000× *g* for 10 min, with the supernatant discarded. The remaining cell sediment was resuspended in 1.5 mL of pre-chilled 5 mM MgSO_4_, agitated lightly for 10 min on ice prior to centrifugation at 4 °C, 10,000× *g* for 10 min with the resultant supernatant designated as periplasmic fraction [59].

For the release of intracellular protein fraction, sonication was applied to the sediment obtained, following resuspension in 500 μL of citrate-phosphate buffer, pH 6, on ice for 30 min. In 15 mL centrifuge tubes immersed in ice, sonication proceeded with amplitude set at 50%, pulse of 0.5 s for a duration of 5 min [60]. Centrifugation then proceeded at 4 °C, 16,128× *g* for 10 min with the resultant supernatant designated as cytoplasmic fraction. Finally, insoluble proteins were isolated by the addition of 1 mL of 6 M urea to the resultant sediment and left for 16 h at room temperature prior to centrifugation at 4 °C, 16,128× *g* for 10 min with the resultant supernatant designated as insoluble cytoplasmic fraction.

### 3.10. Determination of Protein Content and β-Glucosidase Activity

For determination of protein content within all fractions, 0.02 mL of each sample was mixed with 1 mL of Bradford reagent (20% (*w*/*v*)) comprised of Coomassie Brilliant Blue G-250 (Fisher Scientific, Waltham, MA, USA), dissolved in methanol, 20% (*v*/*v*) of 85% phosphoric acid, diluted with distilled water and left to stand at room temperature for 2 min. Optical density was subsequently measured at a wavelength of 595 nm, with values compared to known bovine serum albumin standards [61].

Crude samples were then examined using sodium dodecyl sulfate-polyacrylamide gel electrophoresis (SDS-PAGE) [62] with positive samples subjected to further β-glucosidase activity analyses [63] on ρNPG (Merck, Kenilworth, NJ, USA) and glucose of several concentrations. The assay proceeded by incubation of samples with 0.15 g/L of ρNPG in citrate-phosphate buffer at pH 6, in a total volume of 2.2 mL at 40 °C for 30 min. The reaction was ceased by the addition of 2 mL of 1 M Na_2_CO_3_, with the released ρ-nitrophenol content determined at 400 nm. A one-way ANOVA test was conducted for statistical analysis where a value of *p* < 0.05 was considered significantly different. Sample activity on glucose concentrations of 0.05 to 0.25 M was done under the same conditions as described.

## 4. Conclusions

We described here the isolation, characterization, and heterologous expression of novel GH1 β-glucosidase from *T. asperellum* UPM1. Using several in-silico analyses, such as conserved motif identification, physicochemical characterization, phylogeny tree construction, protein localization, and higher structures prediction, a correlation between enzyme sequence and structure to resultant function was deduced. Owing to expected glucose tolerance, experimental works have subsequently proven the function of TaBgl2, with relative activity comparable to previous known protein expression. This work therefore demonstrates the plausibility of comparing novel β-glucosidases to prior homologs for the preliminary assessment of industrial application.

## Figures and Tables

**Table 1:** Prediction of secondary structures within β-glucosidases of selected *Trichoderma* spp., using ExPASY’s GOR IV.

Description	Accession Number	GOR IV Analyses		
		α-Helix (Hh) (%)	Extended Strand (Ee) (%)	Random Coils (%)
β-Glucosidase 2 (*Trichoderma asperellum*) *	ARW78142.1	26.45	18.92	54.62
Glycoside hydrolase family 1 protein (*Trichoderma asperellum* CBS 433.97)	XP_024766195.1	25.38	18.92	55.70
β-Glucosidase (*Trichoderma gamsii)*	XP_018660766.2	27.31	18.28	54.41
Glycoside hydrolase family 1 protein (*Trichoderma atroviride* IMI 206040)	XP_013939543.1	25.81	19.35	54.84
β-1,4-Glucosidase (*Trichoderma virens*)	AJW67427.1	24.84	19.34	55.82
GH1 β-glucosidase BGL2/CEL1a (*Trichoderma guizhouense*)	OPB39337.1	24.30	17.85	57.85
β-Glucosidase (*Trichoderma harzianum*)	KKP02477.1	24.52	17.63	57.85
β-Glucosidase (*Trichoderma reesei*)	BAA74959.1	24.03	18.03	57.94

* This study.

**Table 2:** Physicochemical parameters of β-glucosidases from selected *Trichoderma* spp., computed using ExPASY’s ProtParam tool.

Description	Accession Number	pI	R+	R−	EC (M^−1^·cm^−1^)	II	Stability	AI	GRAVY	Formula	TNA
β-Glucosidase 2 (*Trichoderma asperellum*) *	ARW78142.1	5.55	60	52	102,135	31.25	Stable	69.27	−0.450	C_2395_H_3581_N_631_O_700_S_12_	7319
Glycoside hydrolase family 1 protein (*Trichoderma asperellum* CBS 433.97)	XP_024766195.1	5.45	60	51	102,135	31.25	Stable	69.27	−0.449	C_2393_H_3575_N_631_O_701_S_12_	7312
β-Glucosidase (*Trichoderma gamsii*)	XP_018660766.2	5.23	63	51	103,625	32.33	Stable	68.65	−0.485	C_2397_H_3576_N_630_O_706_S_12_	7321
Glycoside hydrolase family 1 protein (*Trichoderma atroviride* IMI 206040)	XP_013939543.1	5.30	62	51	103,625	31.58	Stable	69.89	−0.464	C_2401_H_3587_N_631_O_705_S_12_	7336
β-1,4-Glucosidase (*Trichoderma virens)*	AJW67427.1	5.53	57	49	102,135	31.80	Stable	68.86	−0.442	C_2338_H_3489_N_619_O_685_S_12_	7143
GH1 β-glucosidase BGL2/CEL1a (*Trichoderma guizhouense*)	OPB39337.1	5.10	64	50	104,990	25.55	Stable	72.19	−0.450	C_2409_H_3589_N_627_O_704_S_11_	7340
β-Glucosidase (*Trichoderma harzianum*)	KKP02477.1	5.11	64	50	104,990	26.23	Stable	71.35	−0.454	C_2409_H_3589_N_627_O_704_S_12_	7341
β-Glucosidase (*Trichoderma reesei*)	BAA74959.1	5.33	60	49	102,010	27.54	Stable	70.60	−0.396	C_2368_H_3534_N_628_O_693_S_11_	7234

* This study; Notes: Isoelectric point (pI), number of positive and negative amino acids (R+/**−**); extinction coefficient (EC); instability index (II); aliphatic index (AI); grand average of hydropathicity (GRAVY); total number of atoms (TNA).

**Table 3:** Amino acid composition (%) of selected *Trichoderma* spp. β-glucosidases computed using ExPASy’s ProtParam tool.

Amino Acid	ARW78142.1 *	XP_024766195.1	XP_018660766.2	XP_013939543.1	AJW67427.1	OPB39337.1	KKP02477.1	BAA74959.1
Ala (A)	7.7	7.7	7.5	7.5	7.7	7.3	7.3	9.0
Arg (R)	5.8	5.8	5.8	5.8	5.9	5.6	5.6	5.8
Asn (N)	4.5	4.7	4.5	4.7	4.8	4.7	4.7	4.1
Asp (D)	7.3	7.3	7.7	7.5	7.3	8.2	8.0	7.9
Cys (C)	1.3	1.3	1.3	1.3	1.3	1.1	1.1	1.1
Gln (Q)	2.8	2.8	2.8	2.8	2.9	2.6	2.6	2.6
Glu (E)	5.6	5.6	5.8	5.8	5.3	5.6	5.8	4.9
Gly (G)	8.0	8.0	8.0	7.7	8.1	8.2	8.2	9.0
His (H)	1.5	1.5	1.5	1.5	1.5	1.5	1.5	1.7
Ile (I)	5.4	5.4	5.8	5.6	5.5	5.2	5.2	5.2
Leu (L)	6.9	6.9	6.7	6.9	6.6	8.0	7.7	6.7
Lys (K)	5.4	5.2	5.2	5.2	4.8	5.2	5.2	4.7
Met (M)	1.3	1.3	1.3	1.3	1.3	1.3	1.5	1.3
Phe (F)	6.5	6.5	6.2	6.2	6.4	6.2	6.2	6.0
Pro (P)	5.6	5.6	5.8	5.6	5.5	5.8	5.8	6.0
Ser (S)	6.0	6.0	5.8	5.6	6.2	5.2	5.2	5.8
Thr (T)	6.0	6.0	6.0	6.2	6.2	5.6	5.6	5.2
Trp (W)	2.6	2.6	2.6	2.6	2.6	2.6	2.6	2.6
Tyr (Y)	5.2	5.2	5.4	5.4	5.3	5.6	5.6	5.2
Val (V)	4.7	4.7	4.3	4.7	4.8	4.7	4.7	5.4

* This study.

**Table 4:** Comparison of hydrophobic scores and positions of selected *Trichoderma* spp. β-glucosidases using ExPASY ProtScale tool.

Description	Accession Number	Position		Score	
		Min	Max	Min	Max
β-Glucosidase 2 (*Trichoderma asperellum*) *	ARW78142.1	445	114	−2.444	1.667
Glycoside hydrolase family 1 protein (*Trichoderma asperellum* CBS 433.97)	XP_024766195.1	445	114	−2.444	1.667
β-Glucosidase (*Trichoderma gamsii*)	XP_018660766.2	445	114	−2.444	1.667
Glycoside hydrolase family 1 protein (*Trichoderma atroviride* IMI 206040)	XP_013939543.1	445	114	−2.444	1.667
β-1,4-Glucosidase (*Trichoderma virens*)	AJW67427.1	445	114	−2.444	1.667
GH1 β-glucosidase BGL2/CEL1a (*Trichoderma guizhouense*)	OPB39337.1	445	195	−2.444	1.689
β-Glucosidase (*Trichoderma harzianum*)	KKP02477.1	445	195	−2.444	1.689
β-Glucosidase (*Trichoderma reesei*)	BAA74959.1	446	114	−2.489	1.667

* This study.

**Table 5:** Codon usage of *Escherichia coli* corresponding to each amino acid and ‘stop’ signal (abbreviated), compared to codons present in native (bgl2n) and codon optimized (bgl2co) TaBgl2 gene sequences.

Amino Acid	Codon	Frequency			Amino Acid	Codon	Frequency		
		*E. coli*	bgl2n	bgl2co			*E. coli*	bgl2n	bgl2co
ILE	ATT	0.49	0.28	0.56	TRP	TGG	1.00	1.00	1.00
	ATC	0.39	0.72	0.44	CYS	TGT	0.46	0.17	0.00
	ATA	0.11	0.00	0.00		TGC	0.54	0.83	1.00
LEU	CTT	0.12	0.16	0.00	ALA	GCT	0.18	0.17	0.00
	CTC	0.10	0.16	0.00		GCC	0.26	0.61	0.00
	CTA	0.04	0.00	0.00		GCA	0.23	0.06	0.00
	CTG	0.47	0.50	1.00		GCG	0.33	0.17	1.00
	TTA	0.14	0.00	0.00	GLY	GGT	0.35	0.14	0.59
	TTG	0.13	0.19	0.00		GGC	0.37	0.57	0.41
VAL	GTT	0.28	0.27	0.50		GGA	0.13	0.24	0.00
	GTC	0.20	0.41	0.00		GGG	0.15	0.05	0.00
	GTA	0.17	0.05	0.00	PRO	CCT	0.18	0.15	0.00
	GTG	0.35	0.27	0.50		CCC	0.13	0.50	0.00
	TTT	0.58	0.47	0.47		CCA	0.20	0.23	0.00
	TTC	0.42	0.53	0.53		CCG	0.49	0.12	1.00
MET	ATG	1.00	1.00	1.00	THR	ACT	0.19	0.18	0.00
STOP	TAA	0.61	1.00	1.00		ACC	0.40	0.36	1.00
	TGA	0.30	0.00	0.00		ACA	0.17	0.11	0.00
	TAG	0.09	0.00	0.00		ACG	0.25	0.36	0.00
PHE	TTT	0.58	0.47	0.47	TYR	TAT	0.59	0.33	0.46
	TTC	0.42	0.53	0.53		TAC	0.41	0.67	0.54
SER	TCT	0.17	0.21	0.00	ARG	CGT	0.36	0.04	1.00
	TCC	0.15	0.21	0.00		CGC	0.36	0.52	0.00
	TCA	0.14	0.07	0.00		CGA	0.07	0.22	0.00
	TCG	0.14	0.21	0.00		CGG	0.11	0.04	0.00
	AGT	0.16	0.04	0.00		AGA	0.07	0.15	0.00
	AGC	0.25	0.25	1.00		AGG	0.04	0.04	0.00
GLN	CAA	0.34	0.15	0.31	ASP	GAT	0.63	0.44	0.47
	CAG	0.66	0.85	0.69		GAC	0.37	0.56	0.53
ASN	AAT	0.49	0.14	0.00	LYS	AAA	0.74	0.24	0.40
	AAC	0.51	0.86	1.00		AAG	0.26	0.76	0.60
HIS	CAT	0.57	0.14	0.00	GLU	GAA	0.68	0.27	0.42
	CAC	0.43	0.86	1.00		GAG	0.32	0.73	0.58

**Table 6:** Degenerate primers from the conserved regions of β-glucosidase amino acid sequence variants from *Trichoderma* sp. belonging to the Bgl2/Cel1A family.

Primer	Conserved Region	Sequence (5′ to 3′)
DGFAMILYF	YQIEGA	TAYCARATHGARGGNGC
DGFAMILYR	DFYGMN	TTCATNCCRTARAARTC

**Table 7:** Gene specific primers designed for rapid amplification of cDNA ends (RACE) PCR. 5′-GATTACGCCAAGCTT-3′ sequence introduced and highlighted in bold, is for cloning into vector, as suggested by SMARTer ^®^ RACE 5′/3′ Kit User Manual.

Primer	Sequence (5′ to 3′)
GSPFAM1F	**GATTACGCCAAGCTT**GACGACCTGCTGGAAGCGGGCATCACC
GSPFAM1R	**GATTACGCCAAGCTT**ATCGCTGGTGGTCTTGAACTCCTCTCG

## Supplementary Materials

Supplementary materials can be found at https://www.mdpi.com/1422-0067/21/11/4035/s1.

## Abbreviations

Bgl	Beta-glucosidase
ANOVA	Analysis of Variance
GH	Glycoside hydrolase
ρNPG	ρ-nitrophenyl-β-d-glucopyranoside
CAZy	Carbohydrate-Active Enzymes
GH1	Glycoside hydrolase family 1
GH3	Glycoside hydrolase family 3
BLAST	Basic Local Alignment Search Tool
ORF	Open reading frame
MSA	Multiple sequence alignments
TIM	Triosephosphate isomerase
pI	Isoelectric point
GRAVY	Grand average of hydropathicity
PDA	Potato dextrose agar
CMC	Carboxymethylcellulose
RACE	Rapid amplification of cDNA ends
EDTA	Ethylenediaminetetraacetic acid
SDS-PAGE	Sodium dodecyl sulfate-polyacrylamide gel electrophoresis
