# Supplementary material for: In-Silico Characterization of Glycosyl Hydrolase Family 1 β-Glucosidase from Trichoderma asperellum UPM1
Source: Int J Mol Sci. 2020 Jun 4;21(11):4035. doi: 10.3390/ijms21114035 (PMC7311958; doi:10.3390/ijms21114035)
Supplement: Supplementary file 1 [file ijms-21-04035-s001.pdf]

Supplementary Materials

Table S1. Comparison of nucleotide sequence obtained following rapid amplification of cDNA ends (RACE) PCR using gene specific primers.

Description	Identity (%)	Accession Number
β-Glucosidase 2 (*Trichoderma asperellum*)*	100	ARW78142.1
Trichoderma virens beta-1,4-glucosidase (bgl2) gene, complete cds	98.64	KM052276.1
Trichoderma virens bgl1 (BGL1) mRNA, complete cds	98.61	KU535892.1
Trichoderma asperellum CBS 433.97 glycoside hydrolase family 1 protein	98.57	XM_024909259.1
Trichoderma gamsii beta-glucosidase (TGAM01_v203663), partial mRNA	91.85	XM_018806048.2
Trichoderma atroviride IMI 206040 glycoside hydrolase family 1 protein partial mRNA	91.70	XM_014084068.1

* This study

Table S2. Comparison of predicted TaBgl2 amino acid sequence to selected β-glucosidases from several *Trichoderma* spp.

Description	Length (amino acid)	Molecular Weight (Daltons, D)	Identity (%)	Accession Number
β-Glucosidase 2 (*Trichoderma asperellum*) *	465	52,798.31	100	ARW78142.1
Glycoside hydrolase family 1 protein (*Trichoderma asperellum* CBS 433.97)	465	52,784.24	99	XP_024766195.1
β-Glucosidase (*Trichoderma gamsii*)	465	52,899.28	97	XP_018660766.2
Glycoside hydrolase family 1 protein (*Trichoderma atroviride* IMI 206040)	465	52,956.42	97	XP_013939543.1
β-1,4-Glucosidase (*Trichoderma virens*)	455	51,612.88	99	AJW67427.1
GH1 β-glucosidase BGL2/CEL1a (*Trichoderma guizhouense*)	465	52,950.44	92	OPB39337.1
β-Glucosidase (*Trichoderma harzianum*)	465	52,982.50	92	KKP02477.1
β-Glucosidase (*Trichoderma reesei*)	466	52,240.56	90	BAA74959.1

* This study

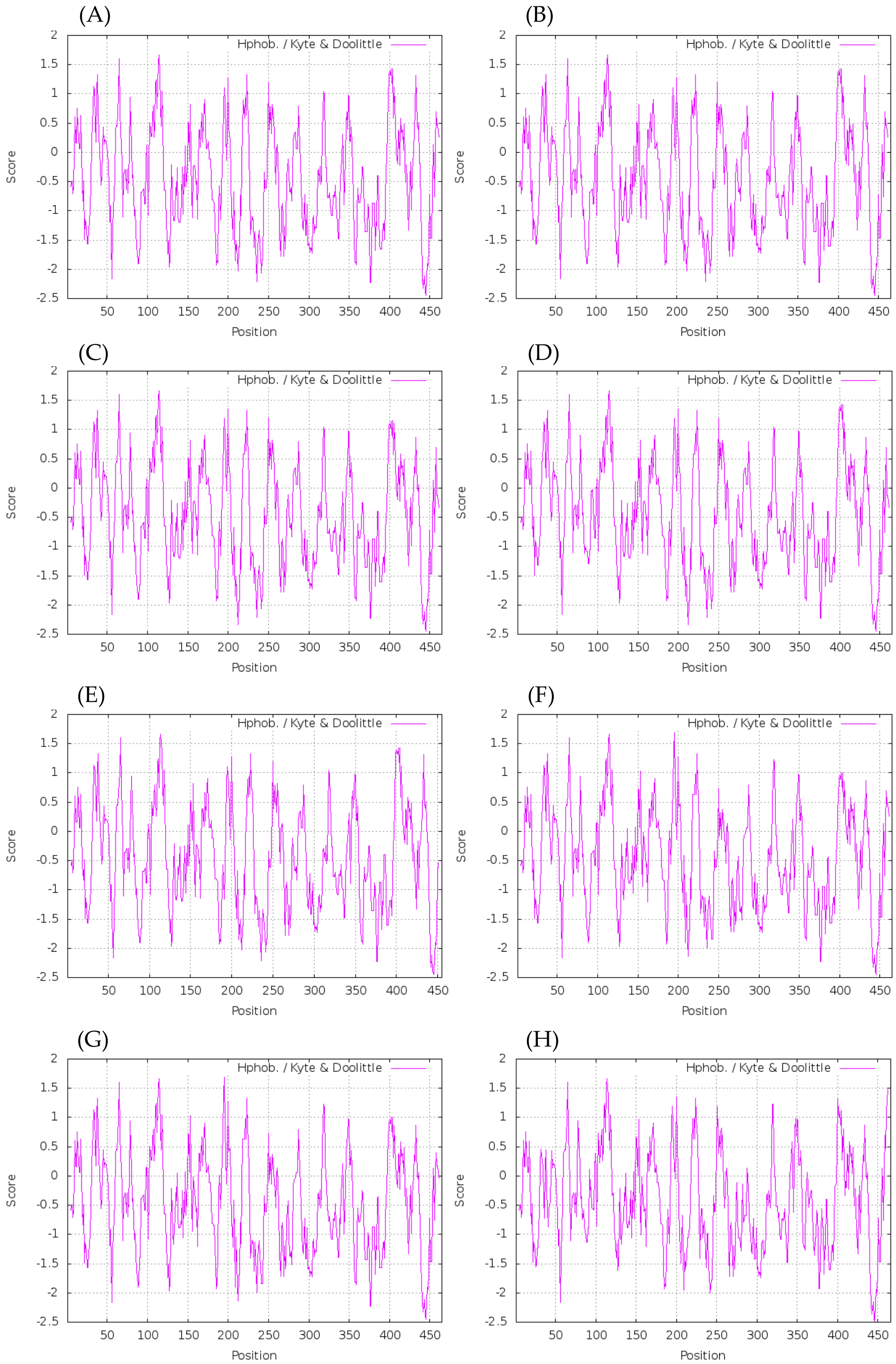

Figure S1. Kyte and Doolittle Plots for β-glucosidases from different *Trichoderma* sp. (A: *T. asperellum* UPM1 Bgl 2 – ARW78142.1; B: *T. asperellum* CBS 433.97 glycoside hydrolase family 1 – XP_024766195.1; C: *T. gamsii* beta-glucosidase – XP_018660766.2; D: *T. atroviride* IMI 206040 glycoside hydrolase family 1 – XP_013939543.1; E: *T. virens* beta-1,4-glucosidase – AJW67427.1; F: *T. guizhouense* GH1 beta-glucosidase Bgl2 – OPB339337.1; G: *T. harzianum* beta-glucosidase – KKP02477.1; H: *T. reesei* beta-glucosidase – BAA74959.1). Dashed lines in red indicate threshold value of 1.6.

Table S3. Subcellular localization of TaBgl2 following prediction from (**A**) PSORT II and (**B**) DeepLoc 1.0 servers (including protein solubility).

(A)

Localization	Probability (%)
Cytoplasmic	56.5
Nuclear	17.4
Cytoskeletal	17.4
Endoplasmic reticulum	4.3
Peroxisomal	4.3

(B)

Localization	Probability	Solubility
Cytoplasm	0.6021	Soluble
Peroxisome	0.1255	
Plastid	0.0810	
Mitochondrion	0.0734	
Lysosome/Vacuole	0.0455	
Extracellular	0.0349	
Endoplasmic reticulum	0.0226	
Cell membrane	0.0080	
Nucleus	0.0059	
Golgi apparatus	0.0010	
